# Low temperature synthesis of ZnO particles using a CO_2_-driven mechanism under high pressure

**DOI:** 10.1039/d3ra07067k

**Published:** 2024-02-08

**Authors:** Taishi Furuya, Yusuke Shimoyama, Yasuhiko Orita

**Affiliations:** a Department of Chemical Science and Engineering, Tokyo Institute of Technology 2-12-1 S1-33, Ookayama, Meguro-ku Tokyo 152-8550 Japan orita.y.aa@m.titech.ac.jp

## Abstract

Low temperature synthesis of ZnO particles without using reactive materials, solvents and post-treatments is still a serious challenge for both fundamental research and industrial applications. In this research, we report the dry synthesis of ZnO particles only by using Zn(acac)_2_ and supercritical CO_2_ (scCO_2_) at the low temperature of 60 °C. The synthesis was performed using CO_2_ and N_2_ from 0.1 to 30.0 MPa for 18 h. As a result, ZnO yields increased with a rise in the CO_2_ pressure and reached 67% at 30.0 MPa while N_2_ medium gave low yields below 4.9% regardless of the pressure. Additionally, the detailed characterization results and the phase behavior observations evidentially showed the formation of zinc–CO/CO_2_–organic complexes in the solid phase of Zn(acac)_2_ powder under scCO_2_, resulting in the accelerated formation of ZnO particles. These findings suggest that scCO_2_ has potential value to drive the formation reaction of zinc–CO/CO_2_–organic complexes, which allows the low temperature synthesis of ZnO particles under dry conditions without using reactive materials, solvents and post-treatments.

## Abstract 2

Low temperature synthesis of ZnO particles using a new reaction mechanism driven by supercritical CO_2_.

## Graphical abstract

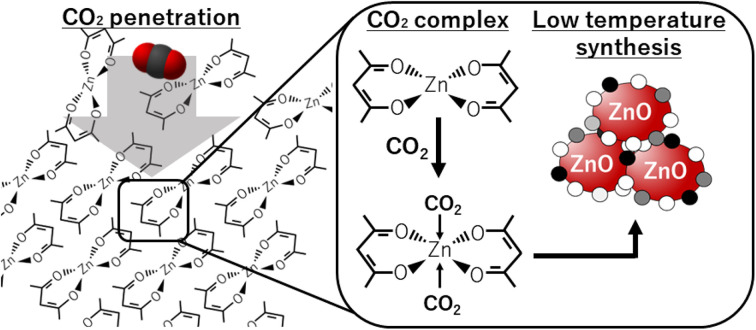

## 1. Introduction

Zinc oxide (ZnO) is utilized in cosmetics,^1,2^ paints,^3^ gas sensors,^4,5^ photocatalysts^6,7^ and drugs^8–10^ due to its low toxicity^11^ and excellent optical and electrical properties such as a wide band gap of 3.37 eV and a high exciton binding energy of 60 meV.^12^ Therefore, ZnO particles have been synthesized using various gas and liquid phase methods, such as chemical vapor deposition,^13^ spray pyrolysis^14^ and plasma,^15^ sol–gel,^16^ precipitation^17^ and hydrothermal methods.^18^ Although the gas phase methods have the advantage of requiring no post-treatment, they require high temperature conditions, leading to the increased cost and limiting applications.^19^ Although the liquid phase methods can allow the synthesis of ZnO particles at low temperature, they cause a large amount of liquid waste for the synthesis and washing.^20^ Therefore, the low temperature synthesis of ZnO particles without a post-treatment is still a serious challenge for the development of a green chemistry approach.

Supercritical CO_2_ (scCO_2_) is known as an environmentally benign medium for the synthesis of inorganic materials due to its nontoxicity, cheapness and recyclability.^21^ Additionally, scCO_2_ has high solubility of metal organic precursor and high diffusivity while the synthesis in scCO_2_ is substantially solventless process because scCO_2_ is easily removed only by reducing the pressure.^22,23^ Furthermore, it is revealed in our recent research that scCO_2_ can be used as not only synthesis medium but also washing and drying solvents for the particle production.^24^ These appealing characteristics enable the simple production process without using an organic solvent and a post-treatment. Therefore, scCO_2_ has been used as a synthesis field of various inorganic particles such as metal,^25–27^ metal oxide^28–31^ and metal sulfide.^32^ In these syntheses, scCO_2_ is typically used as a solvent to dissolve precursors for controlling the reaction, which means that scCO_2_ has no role to drive the reaction and to directly reduce the reaction temperature. Therefore, low temperature synthesis driven by scCO_2_ is a serious challenge for fundamental research and industrial applications.

Similar challenge exists in also the synthesis of ZnO particles from various Zn precursor under scCO_2_. For example, Haldorai *et al.* report the synthesis of ZnO particles from Zn(NO_3_)_2_ under scCO_2_ + ethanol (10 vol%) mixture at 300 °C.^28^ Vostrikov *et al.* report the synthesis of ZnO from metallic and bulk zinc material (prepared by casting melted zinc) under scCO_2_ or scCO_2_ + H_2_O (31.6–82.8 mol%) at the temperatures from 330 to 600 °C.^29^ Whereas Chang *et al.* report the low temperature synthesis of ZnO particles from metallic zinc film of 80 nm using H_2_O (0.3 vol%) as an oxidant in scCO_2_ at 60 °C.^30^ However, this method requires the preparation of a zinc film by sputtering, and the reaction only occurs on the nanofilm surface, resulting in the slight ZnO quantity. As seen in these previous literatures, some problems of high temperature conditions, much oxidant and low productivity are remained in the ZnO synthesis using various Zn precursors under scCO_2_. Therefore, low temperature and highly productive synthesis should be achieved using a new reaction mechanism driven by scCO_2_ without additional oxidant.

In this research, we report the low temperature and highly productive synthesis of ZnO particles from Zn(acac)_2_ powder only by using scCO_2_ at 60 °C and propose a new reaction mechanism driven by scCO_2_. It is noted that Zn(acac)_2_ is powder state and commercially available, which makes it suitable material for highly productive synthesis although the Zn(acac)_2_ is typically converted into ZnO at the high temperature of 200 °C under air atmosphere.^33^ In this work, to demonstrate the potential of scCO_2_, the synthesis was performed under CO_2_ and N_2_ atmosphere at the pressure from 0.1 to 30.0 MPa. Additionally, the detailed characterizations were applied to the washed and no-washed products to investigate the CO_2_-driven reaction mechanism behind the accelerated formation under scCO_2_.

## 2. Experimental

### 2.1 Materials

Zinc acetylacetonate hydrate (Zn(acac)_2_·xH_2_O, *x* = 0–2) (purity > 98%), 2-methoxyethanol (purity > 99.0%) and ethanol (purity > 99.0%) were purchased from Wako Pure Chemical Industries, Ltd, CO_2_ (purity > 99.9%), nitrogen (N_2_, purity > 99.95%) and ultra-high-pressure N_2_ (purity > 99.95%) were supplied by Fujii Bussan Co., Ltd.

### 2.2 Synthesis

**Fig. 1:**
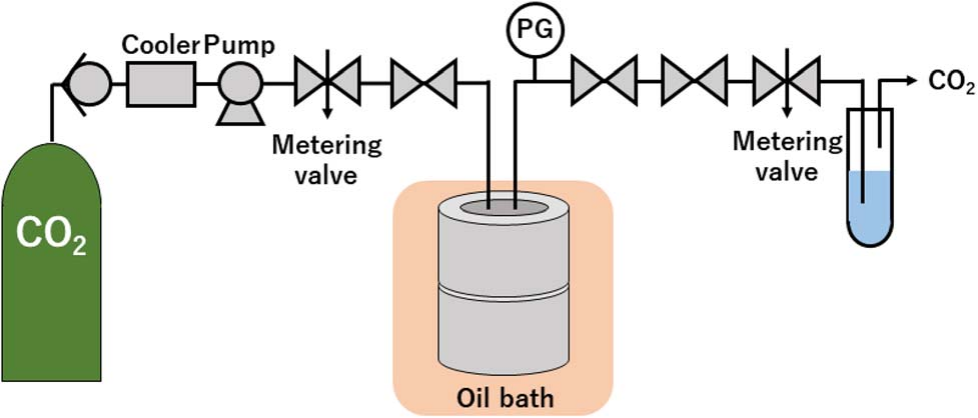
Schematic diagram of experimental apparatus.

The high-pressure system, shown in Fig. 1, was used to synthesize ZnO particles under scCO_2_. After Zn(acac)_2_ of 395 ± 2 mg was loaded into 76 mL reaction vessel (TSC-CO2-008; Taiatsu Glass Corp.), inner air was displaced by flowing CO_2_ for 1 min under 0.5 MPa. Subsequently, liquid CO_2_ was introduced into the vessel using a HPLC pump (PU-4386; JASCO Co., Ltd) until reaching the adequate pressure within 15 min. The inlet and outlet valves were closed and the vessel was sunk in the oil bath at 60 °C, which resulted in the target pressure from 0.1 ± 0.0 to 30.0 ± 0.3 MPa. After keeping for 18 h, the vessel was removed from the oil bath and was depressurized at a rate of approximately 0.5 MPa min^−1^ using a metering valve (1315G2Y; HOKE Inc.). The experiments were also performed at N_2_ of 0.1 MPa and 30.0 MPa, where N_2_ cylinder was connected to the middle point between the pump and the metering valve. After N_2_ was introduced into the vessel using the metering valve until reaching the arbitrary pressure, the vessel was sunk in the oil bath, which resulted in the target pressure of 0.1 ± 0.0 and 30.0 ± 1.0 MPa. Other conditions and procedures are the same as the synthesis using scCO_2_. It is noted that the error values of weight and pressure represent the maximum or minimum deviations from the set values for all experiments.

The products were washed using several cycles of sonication-centrifugation-decantation with 2-methoxyethanol (360 g) and ethanol (20 g). The final precipitates were dried in a vacuum oven at 22 °C.

### 2.3 Phase observation

The phase behavior was directly observed using a high pressure vessel (volume: 36 mL, Taiatsu Glass Corp.) with sapphire windows. The Zn(acac)_2_ of 187 ± 6 mg was enclosed in the vessel, where the weight against the reactor volume was equal to the above synthesis condition. Subsequently, liquid CO_2_ was introduced into the vessel until reaching the target pressure of 30.0 ± 0.4 MPa. The inside temperature was kept at 60 °C using a mantle heater for 18 h. Other conditions and procedures are the same as the synthesis of ZnO particles using scCO_2_.

### 2.4 Characterization

The particle yield *Y* was defined as follows.1
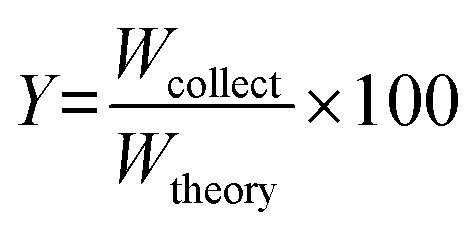
where *W*_collect_ is the weight of products after washing and drying and *W*_theory_ is regarded as the weight of ZnO when anhydrous Zn(acac)_2_ (263 g mol^−1^) is completely converted to ZnO. The products were analyzed by X-ray diffractometry (XRD) (MiniFlex600-C; Rigaku Corp.) using Cu Kα radiation and by a Fourier transform infrared spectrometer (FT-IR) (FT-IR4100; JASCO Co., Ltd). The products were observed by transmission electron microscopy (TEM) (H-7650; Hitachi Corp.) operated at 100 kV. Thermogravimetric (TG) analysis was performed under N_2_ atmosphere using a thermogravimetric analyzer (TGA-50; Shimadzu Corp.). The temperature was increased to 600 °C at a ramp rate of 10 °C min^−1^. Thermogravimetric mass analysis (TG-MS) was performed from room temperature to 500 °C at a ramp rate of 10 °C min^−1^ under He atmosphere using a Thermo plus EVO2 Thermo Mass Photo (Rigaku Corp.). When comparing the relative intensities in each *m*/*z* value, the background spectra at 30 °C was subtracted from original mass spectra to eliminate any background noise.

## 3. Results and discussion

### 3.1 Low temperature synthesis

**Fig. 2:**
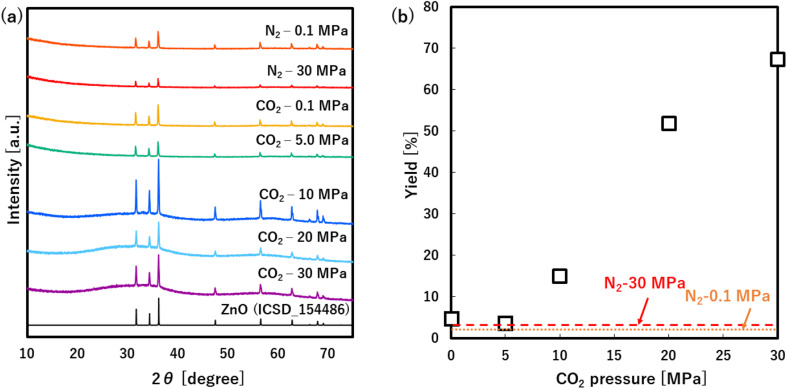
(a) XRD pattern and (b) yield of products synthesized at N_2_ and CO_2_ atmosphere.

As shown in Fig. 2a, all products only showed the hexagonal wurtzite structure of ZnO (ICSD: 154486) regardless of the medium and pressure. Herein, the ZnO yields showed very low values at N_2_ of 0.1 and 30.0 MPa as shown in Fig. 2b, indicating that the Zn(acac)_2_ is not almost converted into ZnO at 60 °C regardless of the pressure. In CO_2_ atmosphere, the yields showed similarly low values at 0.1 and 5.0 MPa, however, remarkably increased with an increase in the pressure from 5.0 to 30.0 MPa. These results clearly indicate that high pressure CO_2_, especially scCO_2_, accelerates the ZnO formation from Zn(acac)_2_, which allows the low temperature synthesis with high yield.

In this system, acetylacetone ligands, CO_2_ and H_2_O (included in Zn(acac)_2_ precursor) are oxygen source. Especially, CO_2_ and H_2_O can be possible oxygen donors for the chemical transformation of Zn(acac)_2_ to ZnO. As detailly mentioned in next section, the no-washed solid product included CO and H_2_O as major components of thermally released material by TG-MS analysis (Fig. 4a and b), which is evident for the possible following reactions using CO_2_ and H_2_O as oxygen donors.

2 Zn(acac)_2_ + CO_2_ + H_2_O → ZnO + 2Hacac + CO + 1/2O_2_

3 Zn(acac)_2_ + H_2_O → ZnO + 2Hacac

The CO_2_ and H_2_O are reported to be oxygen donors in also the previous literature that describe the ZnO synthesis from metallic zinc under scCO_2_.^29,30^ However, it should be emphasized that our system requires only the low temperature of 60 °C and slight water [included in Zn(acac)_2_ precursor] compared to previous system using high temperature above 300 °C^29^ and much water of oxidant.^30^

### 3.2 Analysis of CO_2_-driven mechanism

**Fig. 3:**
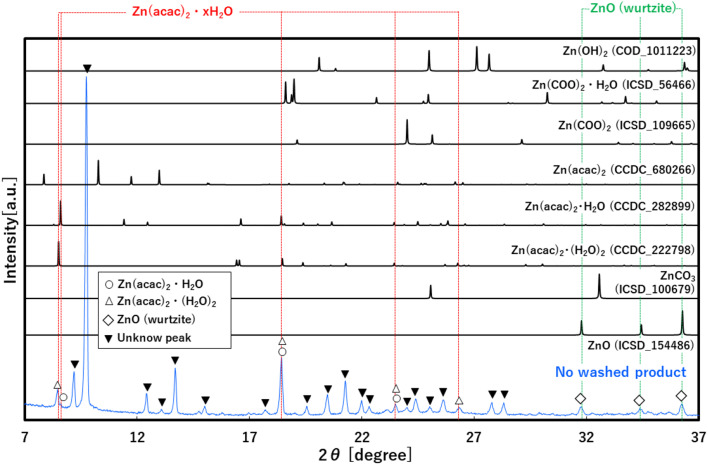
XRD pattern of no-washed product synthesized at 30 MPa of CO_2_ and referenced materials.

**Fig. 4:**
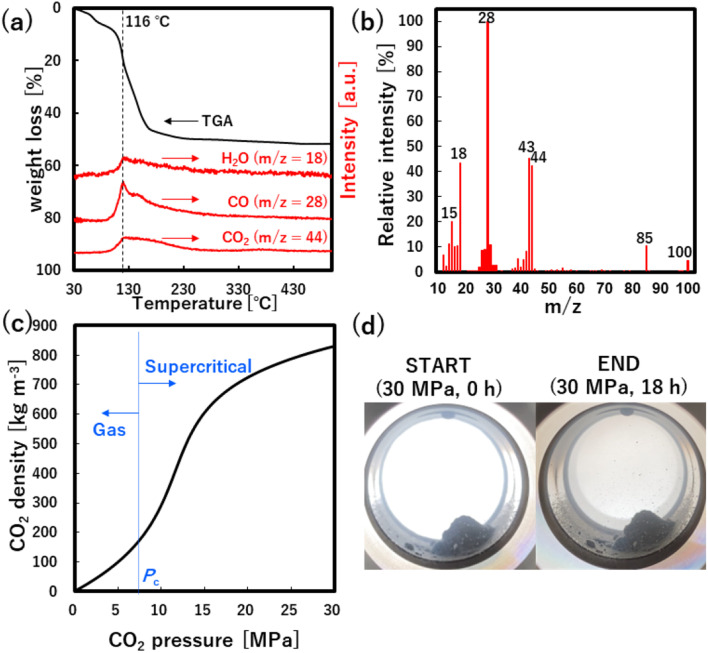
(a) TG-MS results and (b) relative intensity of mass spectra at 116 °C for no-washed product synthesized at 30 MPa of CO_2_. (c) Pressure dependence of CO_2_ density at 60 °C. (d) Phase state at 30 MPa of CO_2_ before and after the reaction.

To investigate the role of CO_2_ behind the accelerated formation, XRD analysis was applied to the no-washed solid product that was synthesized at 30.0 MPa of scCO_2_, as shown in Fig. 3. The XRD pattern showed not only peaks that were assigned to ZnO, Zn(acac)_2_·(H_2_O)_2_ and Zn(acac)_2_·H_2_O, but also many new peaks that were not assigned to the possible byproduct of ZnCO_3_, Zn(COO)_2_, Zn(COO)_2_·H_2_O and Zn(OH)_2_ at the angle position from 7 to 37°. This result suggests the formation of new zinc–organic complexes as intermediate product. To more detailly investigate this complex, TG and TG-MS analyses were applied to the same no-washed product, as shown in Fig. 4a and b. The no-washed product showed the sharp weight loss from 90 to 200 °C for TG analysis while this temperature region resulted in the significant amount of CO (*m*/*z* = 28) and CO_2_ (*m*/*z* = 44) release from no-washed product for TG-MS analysis. In also the mass spectrum at 116 °C, CO and CO_2_ showed strong intensities, where some peaks (*m*/*z* = 15, 18, 43, 85, 100) were assigned to Zn(acac)_2_·H_2_O and its fragments.^34^ These results suggests that scCO_2_ medium leads to the formation of zinc–CO/CO_2_–organic complexes such as Zn(acac)_2_·*x*CO·*y*CO_2_, resulting in the low temperature synthesis of ZnO particles.

Furthermore, the formation of zinc–CO/CO_2_–organic complexes may be related to the CO_2_ density, namely solvation power of CO_2_,^35^ as shown in Fig. 4c. Generally, the solvation power increases with a rise in the CO_2_ pressure and sharply increases above the critical pressure (*P*_c_ = 7.4 MPa), which plausibly accelerates the formation of zinc–CO/CO_2_–organic complexes. Our obtained yields also showed a significant increase above the critical pressure, as shown in Fig. 2b. These results evidentially show that the formation of zinc–CO/CO_2_–organic complexes, namely CO_2_-driven reaction mechanism, allows the low temperature synthesis of ZnO particles with high yield, in addition, its effect significantly increases with a rise in the pressure above the critical point.

The destabilization of Zn precursor by the coordination of CO/CO_2_ (corresponding to the formation of zinc–CO/CO_2_ complex) is one of the possible mechanisms to accelerate the reaction and subsequent the ZnO formation. Yoda *et al.* report the synthesis of Cu from Cu(acac)_2_ dissolved in scCO_2_ at 150–180 °C and the reduction of activation energy by CO_2_ solvation.^36^ Since the coordination is a similar phenomenon to the solvation, the coordination of CO/CO_2_ possibly destabilizes the Zn precursor and reduces the activation energy, allowing the low temperature synthesis under scCO_2_.

However, the formation of zinc–CO/CO_2_–organic complexes seems to occur in not scCO_2_ phase but the solid phase in this work, considering the phase state of enclosed Zn(acac)_2_ powder under scCO_2_ of 30.0 MPa (Fig. 4d). Interestingly, Zn(acac)_2_ powder was not apparently dissolved in scCO_2_ and the piled state of enclosed powder did not change before and after the reaction of 18 h. This result means that zinc–CO/CO_2_–organic complexes and ZnO particles are formed by not the dissolution–precipitation mechanism but the solid phase reaction. ScCO_2_ has unique characteristics of medium solvation power, high diffusivity and small molecular size, which would allow the rapid penetration of CO_2_ into the solid phase, resulting in the formation of zinc–CO/CO_2_–organic complexes and the low temperature synthesis of ZnO particles in the solid phase. Such low temperature synthesis using the formation of zinc–CO/CO_2_–organic complexes in the solid phase has a large potential value for fundamental research since the typical role of scCO_2_ is a solvent to dissolve precursors for the particles production. Furthermore, this system has also a potential value as industrially viable approach with cost-competitiveness since the use of easily available waste heat below 100 °C can minimize the heating cost and solid-phase-like synthesis can allow the mass production due to no requirement of precursor dissolution.

### 3.3 Characterization of ZnO particles

**Fig. 5:**
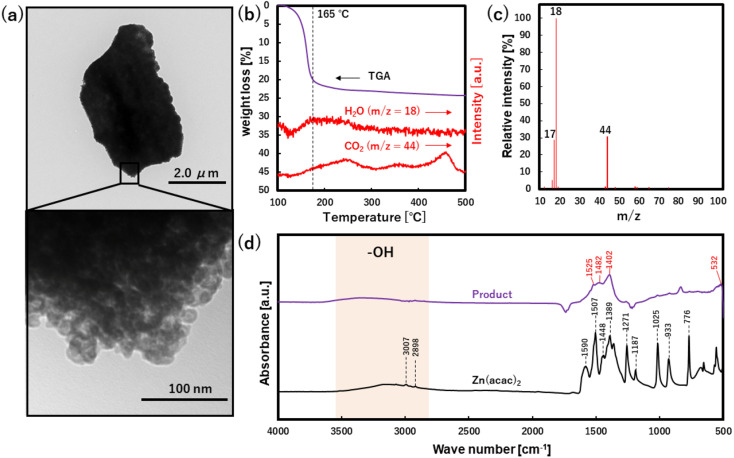
(a) TEM images, (b) TG-MS results, (c) relative intensity of mass spectra at 116 °C and (d) FT-IR spectra for washed product synthesized at 30 MPa of CO_2_.

To characterize the morphology and surface structure of ZnO particles, TEM, TG, TG-MS and FT-IR analyses were applied to the washed product synthesized at CO_2_ of 30.0 MPa, as shown in Fig. 5. The formation of micron-sized aggregates composed of nano-sized primary particles was validated from the TEM images since the gray contrast difference is clearly observed in a partially magnified area of the particle.^37^ Herein, TG analysis showed the weight loss of approximately 20% from 135 to 200 °C (Fig. 5b), where the temperature was kept at 100 °C for 20 min to eliminate the physically absorbed water. Whereas TG-MS analysis, especially the relative intensity of the mass spectrum, showed that OH (*m*/*z* = 17) and H_2_O (*m*/*z* = 18) were the major released components at 165 °C. These results suggest the presence of hydroxyl group on the ZnO surface and crystal water in ZnO bulk structure. It is noted that the CO_2_ (*m*/*z* = 44) had broad multi-peaks from 200 to 600 °C, which may be due to the desorption/thermolysis of some compounds such as oxalate, acetate and amorphous carbon. In the FT-IR analysis, the bands of Zn(acac)_2_ (raw material) were assigned to the stretching vibration of OH (at around 3300 cm^−1^),^38^ the stretching vibration of C–H (at 3007 and 2898 cm^−1^),^38^ the stretching vibrations of C=C, the bending vibrations of C=CH (at 1590 and 1448 cm^−1^),^39,40^ the stretching vibrations of C=O (at 1507 cm^−1^),^41^ the stretching vibrations of C=C or C–CH_3_ (at 1271 and 933 cm^−1^),^39,42^ the rocking of CH_3_ (at 1025 cm^−1^)^43^ and the out-of-plane bending of C–H bonds (at 776 cm^−1^).^42^ However, these bands disappeared or had very low intensities for the product except for broad OH band. Additionally, the product showed the stretching vibration of Zn–O band for the hexagonal wurtzite structure of ZnO (at 532 cm^−1^).^44^ These results further support the presence of hydroxyl group on the ZnO surface. Moreover, the products showed characteristic peaks at the carboxylate-related region from 1525 to 1402 cm^−1^.^43^ It is reported that acetylacetonate bonded to the surface is easily broken and is transformed into the carboxylate group (–COO^−^) due to its unstableness for the formation of ZnO particles.^45^ Therefore, the characteristic peaks at 1525, 1482 and 1402 cm^−1^ would reflect the –COO^−^ bonded to the ZnO surface of primary particles. Additionally, separated three bands suggest the existence of various coordination modes since the separation of symmetric (1402 cm^−1^) and asymmetric (1525 and 1482 cm^−1^) stretching modes for –COO^−^ typically reflects the coordination modes such as unidentate, bridging and bidentate.^46^

### 3.4 Proposed mechanism

**Fig. 6:**
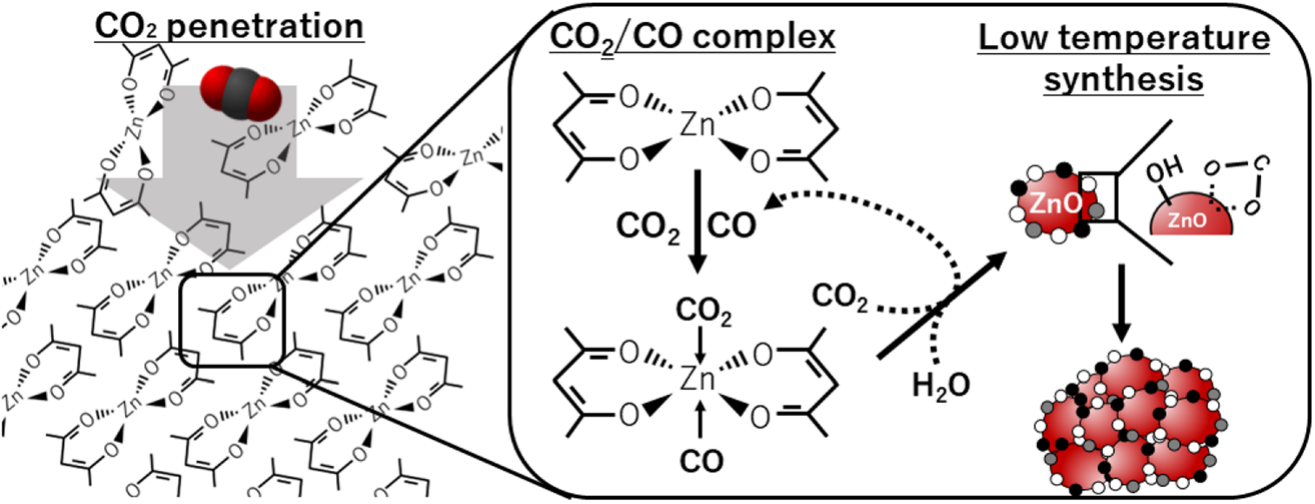
Schematic representation of CO_2_-driven mechanism for the low temperature synthesis of ZnO particles.

Our obtained results can be explained by the following mechanism that is also depicted in Fig. 6. Firstly, scCO_2_, with the characteristics of high diffusivity and small molecular size, penetrates into the solid phase of Zn(acac)_2_. Secondly, the contact of Zn(acac)_2_ with CO_2_ and CO forms zinc–CO/CO_2_–organic complexes in the solid phase, where CO is formed by CO_2_ donates oxygen atom to zinc precursor. Such complex formation allows the low temperature synthesis of ZnO primary particles using CO_2_ and H_2_O as oxygen donors. Finally, ZnO primary particles is aggregated while the acetylacetonate on the ZnO surface is transformed into –COO^−^ due to its unstableness.

## 4. Conclusions

In this research, to investigate the potential of scCO_2_ for the low temperature synthesis of ZnO particles, Zn(acac)_2_ of precursor was contacted with CO_2_ and N_2_ from 0.1 to 30.0 MPa for 18 h at 60 °C. As a result, ZnO yields increased with an increase in the CO_2_ pressure and reached 67% at 30.0 MPa, while N_2_ medium yielded less than 4.9% regardless of the pressure. Additionally, XRD, TG and TG-MS analyses and the direct observations of phase state evidentially showed the formation of zinc–CO/CO_2_–organic complexes in the solid phase of Zn(acac)_2_ powder under scCO_2_, which allowed the low temperature synthesis of ZnO particles. These findings suggest that scCO_2_, with medium solvation power, high diffusivity and small molecular size, has a new potential value to drive the formation of zinc–CO/CO_2_–organic complexes, which allows the low temperature synthesis of ZnO particles under the dry condition without the use of reactive materials, solvents and post-treatments.

## Conflicts of interest

The authors declare no competing financial interest.

## Supplementary Material
